# Functional approach and agro‐climatic information to improve the estimation of olive oil fatty acid content from near‐infrared data

**DOI:** 10.1002/fsn3.1312

**Published:** 2019-12-05

**Authors:** María Isabel Sánchez‐Rodríguez, Elena M. Sánchez‐López, Alberto Marinas, Francisco José Urbano, José M. Caridad

**Affiliations:** ^1^ Statistics and Business Department University of Cordoba Cordoba Spain; ^2^ Organic Chemistry Department University of Cordoba Cordoba Spain

**Keywords:** agro‐climatic curves, extra virgin olive oil, functional data analysis, NIR spectra, regression models

## Abstract

Extra virgin olive oil (EVOO) is very appreciated by its taste, flavor, and benefits for health, and so, it has a high price of commercialization. This fact makes it necessary to provide reliable and cost‐effective analytical procedures, such as near‐infrared (NIR) spectroscopy, to analyze its traceability and purity, in combination with chemometrics. Fatty acids profile of EVOO, considered as a quality parameter, is estimated, firstly, from NIR data and, secondly, by adding agro‐climatic information. NIR and agro‐climatic data sets are summarized by using principal component analysis (PCA) and treated by both scalar and functional approaches. The corresponding PCA and FPCA are progressively introduced in regression models, whose goodness of fit is evaluated by the dimensionless root‐mean‐square error. In general, SFAs, MUFAs, and PUFAs (and disaggregated fatty acids) estimations are improved by adding agro‐climatic besides NIR information (mainly, temperature or evapotranspiration) and considering a functional point of view for both NIR and agro‐climatic data.

## Graphical abstract

Fatty acids, the major components of olive oils, are considered as a quality attribute in the trade standard, and their determination is crucial in studies of traceability and authenticity of oils to prevent frauds. The practical application of this study is centered in the improvement of the estimation of the fatty acids profile of EVOO from NIR data by adding agro‐climatic information (such as temperature or evapotranspiration) in the regression model and considering a functional approach for both NIR and agro‐climatic curves (whose information is summarized by FPCA). The results are applied and highlighted for aggregated (SFAs, MUFAs, and PUFAs) and disaggregated (palmitic, stearic, palmitoleic, oleic, linoleic, and linolenic) fatty acids.

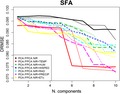

## 1 INTRODUCTION

Extra virgin olive oil (EVOO) is an edible oil highly appreciated by its perfect balance of aroma, taste, and beneficial health properties. Mediterranean countries and Portugal cover 90% of the world production, where Spain and Italy are the major consumers and producers. Andalusia accounts for 80% of the Spanish product. EVOO is obtained only from the olive by mechanical processes only in order that its features are not affected. It is considered as a superior quality oil and so is high‐priced on the market, which makes it susceptible to adulteration with other cheaper oils, such as sunflower, hazelnut, maize, soybean, or refined olive oils (Gurdeniz & Ozen, 2009; Öztürk, Yalçin, & Özdemir, 2010) or indication of untrue geographical origin. For this reason, the European Union has adopted some legislation about Protected Designation of Origin (PDO) and Protected Geographical Indications (PGI) (Aparicio‐Ruiz, García‐González, Lobo‐Prieto, & Aparicio, 2019). Classical chemical separation techniques, such as gas chromatography (GC), use continuous information and need derivatization of sample, with proper integration of separated peaks, to predict the oil content in various compounds. On the contrary, spectroscopic techniques—such as near‐ or mid‐infrared (NIR or MIR) or Fourier transform Raman (FT‐Raman) spectroscopy—generate continuous information too, but derivatization is not necessary, being reliable, rapid, and cost‐effective. IR and Raman spectroscopies can be considered as complementary techniques in the identification of unknown substances in chemical samples. But, an advantage of IR over Raman is the cost because Raman spectroscopy needs high‐powered lasers and amplification sources to get sensitive results, and even, the intense laser radiation can destroy the sample. Besides, IR spectroscopy has been an understood established technique for much longer, so IR provides a greater sensitivity and reliability than Raman techniques. Moreover, in the comparison of NIR vs. MIR, NIR requires more flexible sampling arrangements and cheaper, rugged instrumentation than MIR. Definitely, the utility of NIR is highlighted. The application of multivariate statistics to NIR spectra allows to obtain qualitative or quantitative information of EVOO (Berrueta, Alonso‐Salces, & Héberger, 2007), being useful to avoid fraudulent practices in the oil sector.

The composition of oil is related to the distribution and the type of the fatty acids present in the triglycerides and on the positions in which they are esterified to hydroxyl groups in glycerol backbone. Fatty acids of vegetable oils, considered as a quality parameter, are classified into saturated (SFAs—such as palmitic, myristic, margaric, heptadecanoic, stearic, arachidic, behenic, and lignoceric acids), monounsaturated (MUFAs—such as palmitoleic, margaroleic, heptadecenoic, oleic, eicosenoic, and gadoleic acids), and polyunsaturated (PUFAs—such as linoleic and linolenic, and free fatty acids) fatty acids. The EU Commission Delegated Regulation (2016) and the International Olive Council (2012) consider the fatty acid composition among the characteristics of purity and quality being applicable to olive oils.

There are many studies in the literature of the application of chemometrics to EVOO NIR spectra, specially, with the main aim of its authentication and evaluation of quality parameters. These works show how NIR spectra contain useful and valuable information about EVOO. For instance, NIR spectra have been used for the determination of geographical origins, Protected Designations of Origin (PDO), or compositions (mainly, the fatty acid profile) (Bertran et al., 2000; Casale et al., 2012; Galtier et al., 2007; Mailer, 2004; Sánchez‐Rodríguez et al., 2013, 2014; Woodcock, Downey, & O’Donnell, 2008).

Moreover, there are many works analyzing the influence of weather, agro‐climatic, or meteorological conditions on food content, in general, such as berries (Yang, Laaksonen, Kallio, & Yang, 2017), castor beans (Falasca, Ulberich, & Ulberich, 2012), currants (Zheng et al., 2012), grapes (Luciano, Albuquerque, Rufato, Miquelluti, & Warmling, 2013), mangos (Rymbai et al., 2014), sweet potatoes (Edmunds, Clark, Villordon, & Holmes, 2015), pineapples (Dorey, Fournier, Léchaudel, & Tixier, 2016), or wheat (Khokhar et al., 2017). In particular, many papers treat the effect of these agro‐climatic conditions on olive oils (Awan, 2014; Ozdemir, 2016; Veizi, Peçi, & Lazaj, 2016; Zaied & Zouabi, 2016). But there are few works considering NIR data to study this agro‐climatic influence on oils or other food products. And, in relation to the multivariate statistical technique that has been applied, all the previous studies consider a non‐numerical variable (i.e., a factor) to differentiate among agro‐climatic or meteorological groups. This factor can be subsequently used, for example, as an independent variable in an analysis of variance model or as a dependent variable in linear discriminant analysis. Nevertheless, this paper uses the complete agro‐climatic database obtained from the official webpage of the Automatic Weather Stations (AWSs) of Andalusia, instead of clustering the information in groups. More specifically, the historical daily information has been downloaded, from 2005 to 2010, for the following variables: temperature, humidity, wind speed, radiation, precipitation, and evapotranspiration.

Furthermore, functional data analysis (FDA) is a relatively recent statistical method concerned with the analysis to any data set that can be thought of as a function or a curve (i.e., an infinite‐dimensional variable). FDA was initially popularized by Ramsay and Silverman (2007), and it is actually one of the most active fields of investigation in data science, in general (Aneiros, Cao, Fraiman, Genest, & Vieu, 2019). In particular, the potential of FDA to characterize, compare, and classify chemical data has been analyzed by Burfield, Neumann, and Saunders (2015). But, although FDA has been applied to some examples of NIR data (Aguilera, Escabias, Valderrama, & Aguilera‐Morillo, 2013; Saeys, De Ketelaere, & Darius, 2008), in no case olive oils data were treated by using this approach.

The aim of this work was to determine the profile in fatty acids of EVOO from NIR spectral data, in a first step, and to analyze whether the goodness of fit of the estimation can be improved by also considering agro‐climatic data. Contrary to previous works analyzing and interrelating such sets (Sánchez‐Rodríguez, Caridad, Sánchez‐López, Marinas, & Urbano, 2019; Sánchez‐Rodríguez, Sánchez‐López, Caridad, Marinas, & Urbano, 2018), NIR and agro‐climatic information are contemplated from both scalar and functional points of view. The high‐dimensional data are summarized by using scalar (PCA) and functional (FPCA) principal component analysis. The corresponding PCA and FPCA components are introduced as regressors in models with the fatty acid profile obtained by gas chromatography (GC, classical reference technique) as response. Although many works establish or fix the number of (F) PCA components to be retained, the criteria are usually empirical and nonunanimously accepted. That is why, in this work, PCA and FPCA components are progressively introduced in the models. The reliability of these regression models is compared by using the dimensionless root‐mean‐square error (DRMSE), taking into account the scalar or functional approach of data and the number of retained components (considering the recommendations in the literature respect to the optimal number of components to avoid overfitting (Hawkins, 2004)). Finally, estimations for some disaggregated fatty acids (in particular, palmitic, stearic, palmitoleic, oleic, linoleic, and linolenic) are also determined as the trade standard of olive oil is established based on particular fatty acids.

## 2 MATERIALS AND METHODS

### 2.1 Data

#### 2.1.1 Chemical data

**Figure 1:**
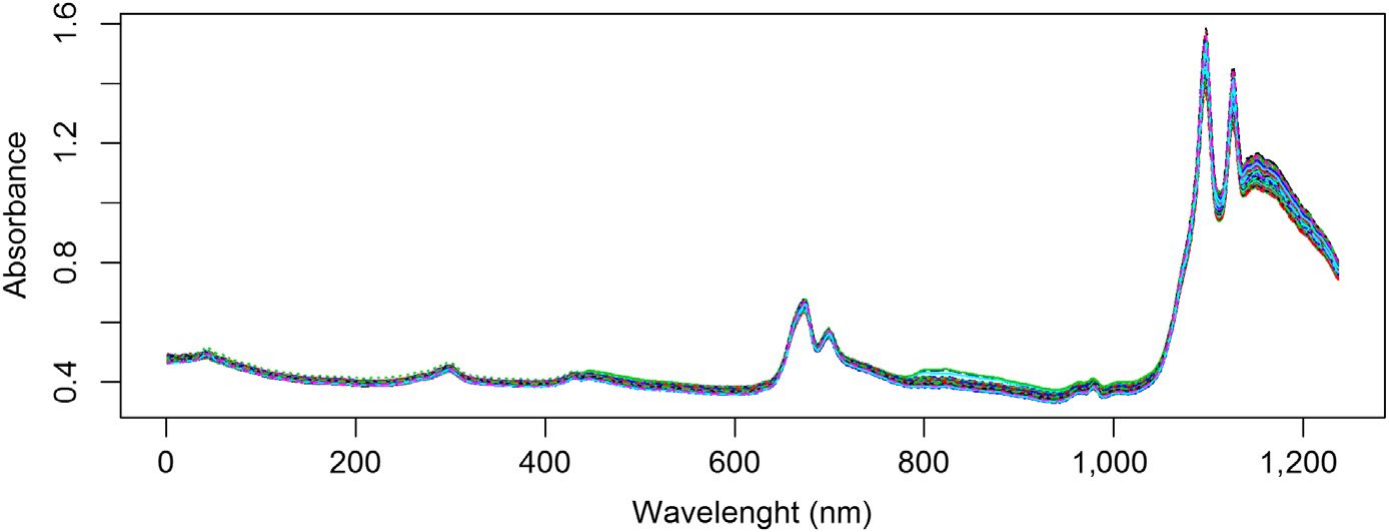
NIR spectra of EVOO

This study is based on data obtained from 222 Andalusian EVOOs collected from 2005 to 2010. Olive oil was either extracted by the producers through a two‐phase centrifugation system or extracted by the staff of the Agronomy Department of University of Cordoba with an Abencor System (which reproduces the industrial process on the laboratory scale and follows the same stages of grinding, beating, centrifugation and decantation). Samples were kept in the fridge in order to their properties were not modified (Baeten, Aparicio, Marigheto, & Wilson, 2003). Within 15 days after reception of the oil samples by the Organic Chemistry Department of the University of Cordoba, NIR spectra were obtained at the Central Service of Analyses (SCAI), also at the University of Cordoba. The instrument employed for spectra collection was a Spectrum One NTS FT‐NIR spectrophotometer (Perkin Elmer LLC, Shelton) equipped with an integrating sphere module. Samples were analyzed by using a transflectance with a glass petri dish and a hexagonal reflector with a total transflectance pathlength of about 0.5 mm. A diffuse reflecting stainless steel surface placed at the bottom of the cup reflected the radiation back through the sample to the reflectance detector. The spectra were obtained with Spectrum Software 5.0.1, and the reflectance (log 1/R) spectra were collected with two different reflectors. Data correspond to the average of results with both reflectors in order to rule out the influence of them on the variability of the obtained results. Furthermore, spectra were afterward smoothed by using the Savitzky and Golay (1964) technique (that performs a local polynomial least squares regression in order to reduce the random noise of the instrumental signal). 1,237 Pretreated NIR data for each olive oil (representing energy absorbed at 1,237 different wavelengths, from 800.62 to 2,499.64 nm) were provided to the Department of Statistics of the University of Cordoba to be analyzed. NIR spectra corresponding to the observed 222 EVOO are shown in Figure 1.

The determinations of GC‐FID fatty acid composition were performed by the staff of Organic Chemistry of University of Cordoba, according to the official methods for olive and pomace oil established by the European Union Commission (2011) and the International Olive Council (2001a, 2001b). The instrument employed was an Agilent 7890A gas chromatograph with a capillary column (SGE FORTE BPX‐70 de 50 m × 220 μm × 0.25 μm), with the following conditions of analysis: 250 ºC of injector temperature, 2 *μ*L of injection volume, and 260 ºC of detector temperature. The oven temperature was programmed to remain at 180 ºC for 15 min and then raised to 240°C at a rate of 4°C/min and maintained at this temperature for 5 min. The triacylglycerol samples (olive oil samples) were submitted to a cold transesterification procedure to convert the triacylglycerol into fatty acid methyl esters. This method is indicated for edible oils with an index of acidity lower than 3.3º: Firstly, 0.1 g of olive oil is transferred into a 5‐mL volumetric flask; secondly, 2 ml *n*‐heptane and 0.2 ml of a 2N KOH solution in methanol were added, and the reaction mixture was vigorously stirred; finally, the methyl esters were extracted and subject to GC analyses.

The EU Commission Delegated Regulation (2016) and the International Olive Council (2012) establish the characteristics of olive oils to determine purity criteria in order to authentication and avoid adulterations with lower quality oils. Particularly, the limit values for fatty acids are regularly updated taking into account the indications of chemical experts and are shown in Table 1.

**Table 1:** Fatty acid composition (in % m/m methyl esters) as determined by GC

Group	Fatty acid	Carbon number	% m/m methyl esters
SFA	Myristic	C14:0	≤0.03
	Palmitic	C16:0	7.50–20.00
	Heptadecanoic	C17:0	≤0.40
	Stearic	C18:0	0.50–5.00
	Arachidic	C20:0	≤0.60
	Behenic	C22:0	≤0.20
	Lignoceric	C24:0	≤0.20
MUFA	Palmitoleic	C16:1	0.30–3.50
	Heptadecenoic	C17:1	≤0.60
	Oleic	C18:1	55.00–83.00
	Eicosenoic	C20:1	≤0.50
PUFA	Linoleic	C18:2	2.50–21.00
	Linolenic	C18:3	≤1.00

*Source:* International Olive Council (2012).

Abbreviations: MUFA, monounsaturated fatty acids; PUFA, polyunsaturated fatty acids; SFA: saturated fatty acid.

#### 2.1.2 Agro‐climatic data

The Spanish official webpage of the Andalusian Institute of Agricultural, Fisheries, Agrifood, and Organic Production Research and Training (at https://www.juntadeandalucia.es/agriculturaypesca/ifapa/ria/servlet/FrontController) provides the long‐run information registered in the Automatic Weather Stations (AWSs). Therefore, this website has been used to obtain the agro‐climatic data of the study: Historical data can be downloaded once selected the name of the station, the agro‐climatic measurements, and the start and end dates. There are approximately 120 AWSs in all the Andalusian provinces, with a suitable plan of maintenance and an exhaustive review of the records that supply the sensors. This work only contemplates the daily information obtained, from 2005 to 2010 (years previous the corresponding oil harvests), for the 28 AWSs specified in Table 2, selected due to their proximity with the cardinal points of extraction of oils.

**Table 2:** Automatic Weather Stations (AWSs)

Province	Station	Code
Cadiz	Villamartín	1
Cordoba	Adamuz	2
	Baena	3
	Belmez	4
	Cabra	5
	Córdoba	6
	El Carpio	7
	Hinojosa del Duque	8
	Hornachuelos	9
	Palma del Río	10
	Santaella	11
Granada	Loja	12
	Pinos Puente	13
Jaen	Alcaudete	14
	Chiclana de Segura	15
	Jaén	16
	Higuera de Arjona	17
	Mancha Real	18
	Marmolejo	19
	Pozo Alcón	20
	San José de los Propios	21
	Santo Tomé	22
Malaga	Antequera	23
	Archidona	24
	Pizarra	25
	Sierra de Yeguas	26
Seville	Écija	27
	Osuna	28

Information about the following variables has been downloaded from each AWS: *Temp*, daily average temperature (ºC); *Hum*, daily average relative humidity (%); *WSpe*, daily average wind speed (m/s); *Rad*, daily average radiation (W/m^2^); *Precip*, daily precipitation (L/m^2^); and *ETo*, the evapotranspiration is the loss of dampness (mm/day) of a surface for either direct evaporation or the water loss for perspiration of the vegetation. Technical information about the measuring instruments can also be obtained from the above‐mentioned link.

**Figure 2:**
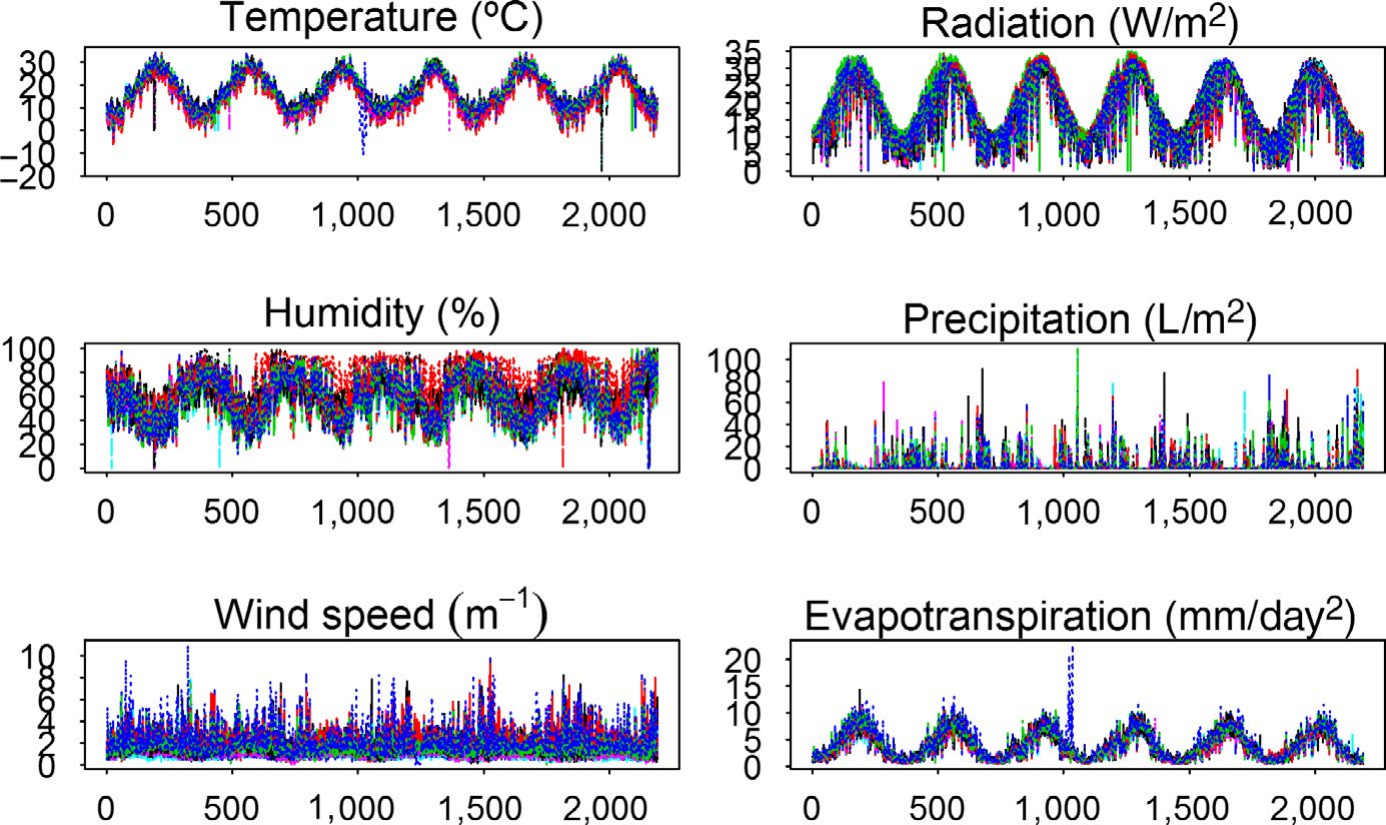
Agro‐climatic spectra for the 28 AWSs

Figure 2 depicts the agro‐climatic series for the observed period (2,191 days, in total, as there is a leap year) and the 28 AWSs. Taking into account the discrepancies among the curves corresponding to the different AWSs, a computer program has been designed by using the R‐project (Team RC, 2018) that permits to associate to each EVOO the agro‐climatic curve corresponding to the year which is preceding to the olives harvest and to the nearest AWS (or the average of the nearest AWSs), for the different six agro‐climatic variables (*Temp*, *Hum*, *WSpe*, *Rad*, *Precip*, and *ETo*). In particular, the programmed R‐function has the following arguments: *station*, *harvest year*, *month1‐month2,* and *agro‐climatic variable* and returns as value the aggregated agro‐climatic measurement according to the previous selection. Detailed information of the R code is included in the Supplementary Material.

**Figure 3:**
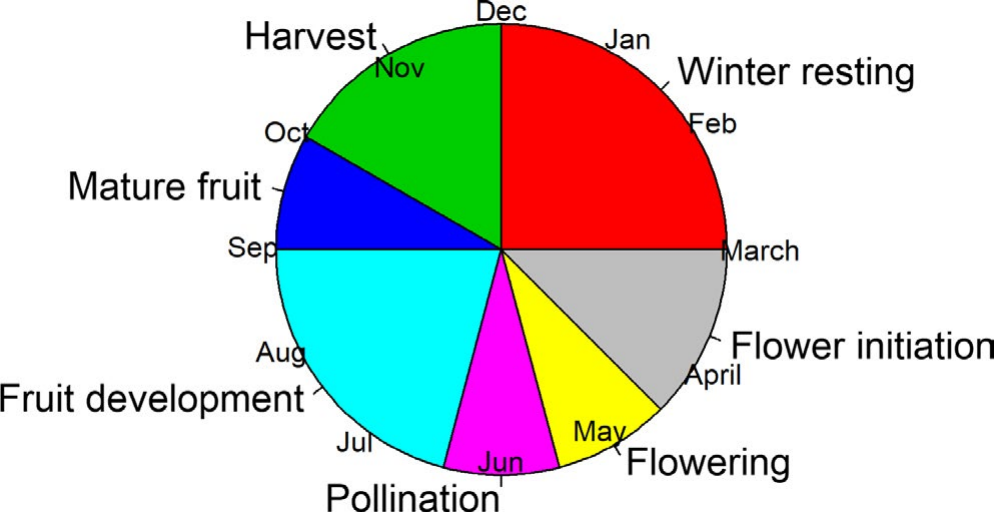
Phenological cycle of the olive grove

Furthermore, the agro‐climatic measurements have been accumulated in order to relate them more adequately to the phenological cycle of the olive grove, which could directly influence the composition of the oil. As shown in Figure 3, this cycle is not equally distributed, and therefore, the months of each period could be studied independently. In the same line, Orlandi, Bonofiglio, Romano, and Fornaciari (2012) study of the influence of climate data on oil production in southern Italy by considering meteorological variables on a monthly basis.

#### 2.1.3 Statistical methodology

NIR and agro‐climatic data provide both huge databases. On one side, NIR spectra associated with each EVOO is the result of measuring the absorbance in more than a thousand wavelengths (1,237). On the other side, six agro‐climatic variables (temperature, humidity, wind speed, radiation, precipitation, and evapotranspiration) can be assigned to each EVOO. Each agro‐climatic series is formed by 2,192 data (corresponding to the daily measurements during six consecutive years, with a leap year).

NIR and agro‐climatic data can be seen either as a scalar view (i.e., as an extensive discretization of points) or as a functional view (i.e., as a curve, observed in an interval). Problems tackled by statistical techniques with functional data are, basically, the same of the classical Statistics (Aneiros et al., 2019). In particular, this work contemplates the fit of regression models to predict the content in fatty acids of EVOO as a function of (scalar or functional) NIR and agro‐climatic data, considering the values obtained by GC as a reference. These models initially contain only NIR information, and then, one of the six agro‐climatic variables is also included among the regressors. All these models are contemplated by a scalar and a functional point of view of the explanatory variables (the response is always scalar), with the aim of comparing the obtained results. The goodness of fit of the models is measured by either RMSE (i.e., the root of the squared differences between observed and estimated values) or DRMSE (obtained by dividing RMSE and the mean of the observed values). DRMSE eliminates the effect of the measurement units and so facilitates the comparison between statistical models. Obviously, the goodness of fit or reliability of models is better as RMSE (or DRMSE) approaches zero.

Moreover, the fact that the number of explanatory variables greatly overcomes the number of cases origins the appearance of multicollinearity (or collinearity). This situation is incompatible with the hypothesis of uncorrelation of general linear models (regression models, in particular) and provides coefficients estimates being unstable before any little change. For that reason, to avoid the presence of multicollinearity, the information contained in (NIR or agro‐climatic) data can be condensed in a few components or latent factors. With this goal, this work uses principal component analysis, a multivariate statistical technique which calculates, from the predictor, a reduced number of components or factors, orthogonal between themselves, by maximizing their internal variance. The above‐mentioned predictors (NIR spectra and agro‐climatic curves) can be considered from a scalar or functional view, resulting principal component analysis (PCA) or the corresponding functional version (FPCA, where the functional data are represented by a orthogonal basis of functional principal components). The objective is not only to reduce the dimensionality of high‐dimensional data sets on a reduced number of components displaying them in a space of a less dimension than but also, fundamentally, to use these PCA and FPCA components to predict the fatty acid profile of EVOO in regression models.

Furthermore, the literature includes many works analyzing the optimal number to be retained in principal component analysis (Saccenti & Camacho, 2015; Vitale et al., 2017). Some of them consider the possibility of progressively including components in the model until one does not significantly increase the explained variability of data. In particular, the classical and ad hoc Kaiser's rule (by default in many statistical software) suggests that those factors explaining a percentage of variability equal or higher than 10 (technically, with eigenvalues equal or higher than 1) should be retained. Some authors do not recommend using this cutoff criterion as it constitutes a case‐specific strategy (not easily generalizable for data of various nature), and it tends to extract too many factors and so over‐extracts components. The overfitting of statistical models is not recommended as it could introduce noise in the regression coefficients and cause some problems in the verification of hypotheses of linear models (Hawkins, 2004). Other more recently developed strategies are based in cross‐validation. These computational criteria are completely data‐driven and distribution‐free, but they sometimes do not discriminate relevant from noisy components and lead to an excessive time and memory consumption. As the most of the established criteria have an empirical character, provided very different results, and are not unanimously applied, this work does not fix the number of factors. Therefore, PCA and FPCA components are progressively introduced in regression models and RMSE is calculated as a function of the number and the type (NIR and agro‐climatic) of the components included in the model, in order to analyze the evolution of the corresponding regression errors. Finally, the results are compared taking into account the recommendations of the literature to avoid the overfitting.

Regarding the software, the R‐project (Team RC, 2018) has been used to connect the databases of NIR and agro‐climatic curves; then, the packages of “pls” (Wehrens & Mevik, 2007), “fda” (Ramsay, Wickham, Graves, & Hooker, 2010), and “fda.usc” (Febrero‐Bande, & Oviedo de la Fuente, 2012) have been used to fit the scalar and functional statistical models. The Supplementary Material contains the code of the R‐project programs.

## 3 RESULTS AND DISCUSSION

In this section, regression models are fit to predict the fatty acids profile of EVOO (obtained by GC as a reference), firstly, as a function of the NIR information and, secondly, when the agro‐climatic daily data are added to the model. NIR spectra and agro‐climatic curves are treated by scalar and functional points of view, being in both cases summarized by principal component analysis (PCA and FPCA, respectively). The goodness of fit of the statistical models is compared by using DRMSE and taking into account de number of components retained in the model.

In order to determine the number of (scalar or functional) principal components to be retained, Table 3 includes this optimal number of components when a classical ad hoc (Kaiser's rule) or a computational (cross‐validation) criterion is considered. Both PCA and FPCA regressions are contemplated, when either only NIR or also agro‐climatic information is added. The results for the classical criterion are different to the results for the actual criterion, but, in general, the results suggest that, in general, to retain more than around ten components could cause overfitting in the regression model. For this reason, the following figures contemplate de‐evolution in DRMSE when PCA and FPCA components, from one to ten, are introduced in the regression models.

**Table 3:** Optimal number of components by considering classical and actual criteria^a^

		N. comp in PCA regression		N. Comp in FPCA regression	
Spectral information		Kaiser's rule	Cross‐validation	Kaiser's rule	Cross‐validation
NIR	NIR	4	7	3	6
NIR + AGR	NIR + TEMP	7	6	7	7
	NIR + HUM	9	10	9	8
	NIR + WSPE	10	11	10	11
	NIR + RAD	8	10	8	10
	NIR + PRECIP	11	10	11	8
	NIR + ETo	10	11	10	9

a In all cases, the percentage of variability of data explained by the selected components is greater than 85%.

**Figure 4:**
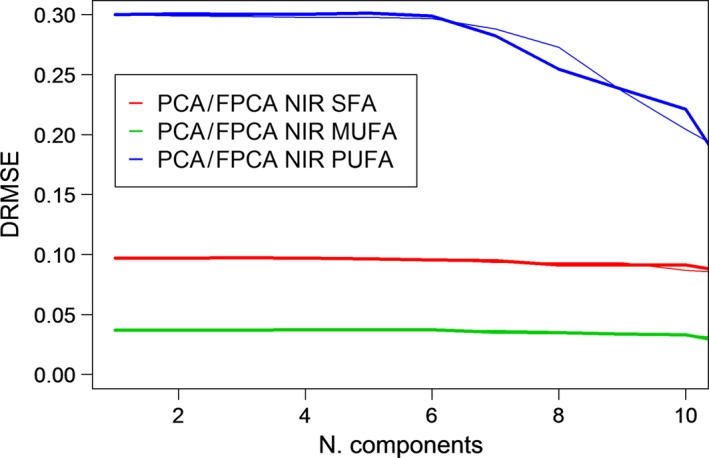
DRMSE in SFA, MUFA, and PUFA estimations by PCA^*^ and FPCA^**^ regression models from NIR data

Figure 4 shows DRMSE in the estimation of SFAs, MUFAs, and PUFAs when NIR spectral information is considered by scalar and functional approaches. The graphics can be overlapped and compared because DRMSE is dimensionless, independent of the units of measurement. The following conclusions can be obtained from the observation of Figure 4:

- The more accurate estimations are obtained for MUFAs (green lines) fatty acids, and less reliable estimations are provided for PUFAs (blue lines). The differences are even more significant when the number of components in the model is low. In fact, DRMSE is quite stable in the estimations of MUFAs and PUFAs, with a slight improvement with the introduction of (PCA or FPCA) components in the models. The situation for PUFAs is the opposite as the estimations have a high corresponding DRMSE initially, and it decreases with the introduction of components in the models.
- For each fatty acid, the goodness of fit of estimations from the PCA and FPCA regression models is quite similar and, so, the treatment of NIR data by a functional view does not improve the estimations obtained by their scalar view when only chemical spectral data are used.

**Figure 5:**
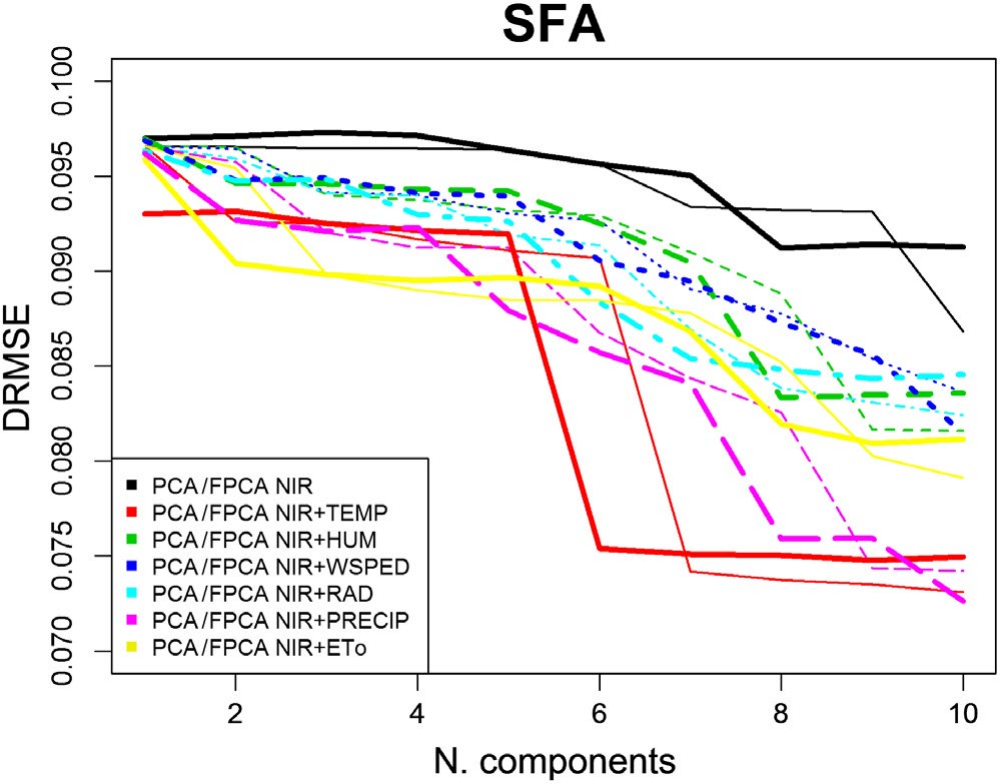
RMSE in SFA estimations by PCA^*^ and FPCA^**^ regression models from NIR and agro‐climatic data

**Figure 6:**
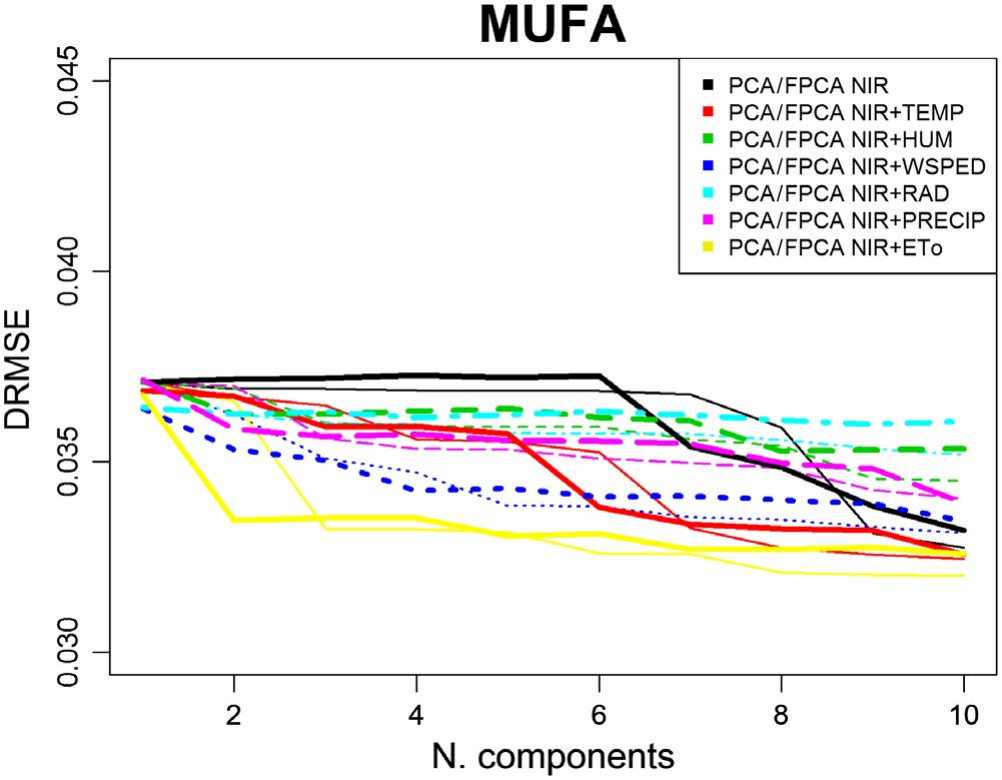
RMSE in MUFA estimations by PCA^*^ and FPCA^**^ regression models from NIR and agro‐climatic data

**Figure 7:**
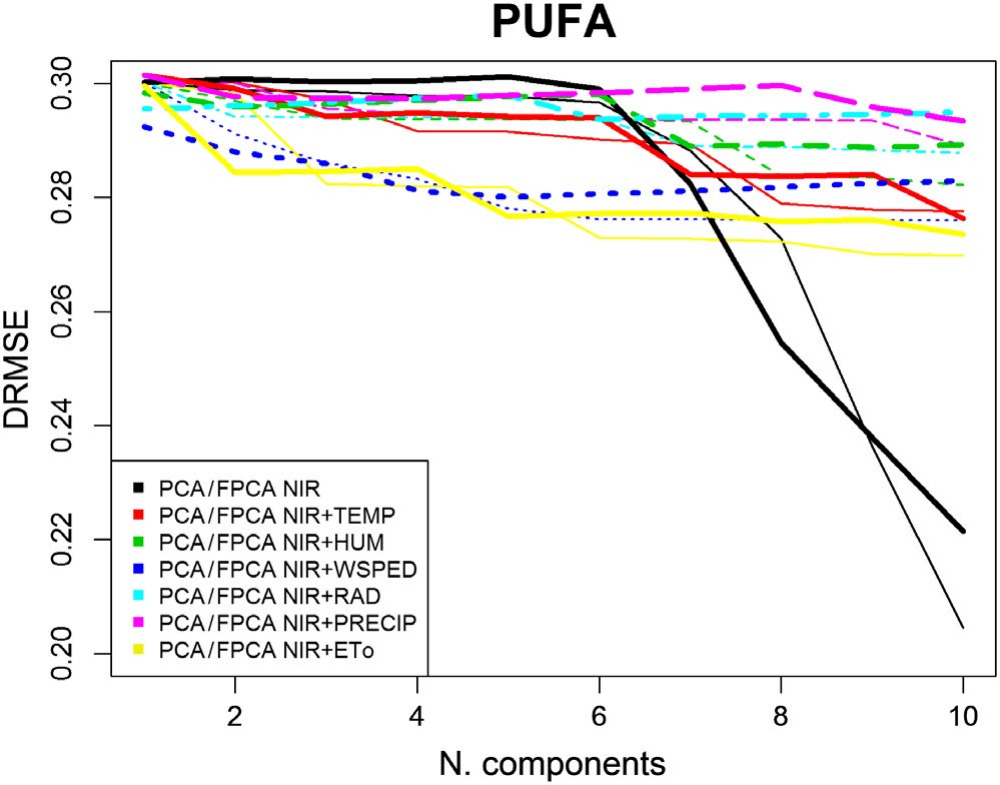
RMSE in MUFA estimations by PCA^*^ and FPCA^**^ regression models from NIR and agro‐climatic data

Figures 5, 6, 7 depict DRMSE in the estimations of SFAs, MUFAs, and PUFAs fatty acids of EVOO by using only NIR spectral information (black lines) or also adding a specific agro‐climatic information (the remaining lines): temperature (red), humidity (green), wind speed (dark blue), radiation (light blue), precipitation (pink), or evapotranspiration (yellow). In each case, both set of data are treated from a scalar (thin lines) or functional (thick lines) point of view in other to fit the corresponding PCA or FPCA regression models. The main conclusions are as follows:

- In general, the goodness of fit of the estimations of SFAs, MUFAs, and PUFAs is better when the agro‐climatic information (in addition to the NIR one) is also included. Only in the estimation of PUFAs fatty acids, when the number of components is progressively increased, the best estimations are obtained from models only containing NIR information.
- Also in general terms, the goodness of fit of the three types of fatty acids is better when data (NIR or agro‐climatic) are treated from a functional approach for a low number of components (in FPCA regression).
- Taking into account, the differences between the type of agro‐climatic information included in the regression models, for a low number of components, evapotranspiration (yellow line), temperature (red line), and precipitation (pink line), are the variables providing the first significant reduction in SFAs DRMSE. In the case of MUFAs and PUFAs DRMSE, the most relevant variables (because of the corresponding decrease in DRMSE) are the evapotranspiration (yellow line) and wind speed (dark blue line).

Finally, Figures [Link], [Link], [Link]–S3 in Appendix (see Supplementary Material) shows DRMSE associated with some disaggregated fatty acids, namely palmitic and stearic (SFAs fatty acids), palmitoleic and oleic (MUFAs), and linoleic and linolenic (PUFAs). Their particular estimation is significant in order to analyze the traceability and purity of olive oils as the limit values for fatty acids established by the EU Commission Delegated Regulation (2016) and the International Olive Council (2012), periodically updated, are fixed for disaggregated (not aggregated in SFAs, MUFAs, and PUFAs) fatty acids. Nevertheless, the aspect of the graphic for the disaggregated acid is quite similar to the corresponding aggregated one. The following conclusions can be obtained:

- In an individual interpretation of the graphics, in general lines, the conclusions for the disaggregated fatty acids are similar to the ones obtained for SFAs, MUFAs, and PUFAs: functional PCA regression models including NIR and agro‐climatic (mainly, temperature, or evapotranspiration) information provide the best goodness of fit. Only in the case of linoleic and linolenic (as for PUFAs fatty acids, in general), the situation is the opposite for a number of components greater than seven–eight (a higher number than recommended to avoid overfitting in Table 3).
- As dimensionless RMSE (DRMSE) are represented in y‐axis with the same range (0 to 0.4), the values for the different graphics can be compared. Although Figure 4 depicts that the most accuracy is associated with MUFAs estimations, there is a great difference in the DRMSE associated with the disaggregated palmitoleic and oleic acids, being better the estimations of the last one. In fact, oleic estimations are the best among all the disaggregated fatty acids estimations. The minor difference exits between SFAs (palmitic and stearic) acids. The estimations for linoleic and linolenic (PUFAs acids) are quite different too.

## 4 CONCLUSIONS

Last years, fast, reliable, and cost‐effective analytical procedures have been established in studies about purity, authentication, and traceability of olive oils. In this sense, NIR spectra have been habitually used, in combination with chemometrics, to determine interesting qualitative and quantitative information about olive oils. Moreover, the literature contains many works analyzing the influence of agro‐climatic conditions on food components, in general, and on olive oils, in particular. But all these works contemplate this agro‐climatic information as a factor, a non‐numerical variable. Furthermore, FDA actually constitutes an active field of investigation in data science, being used with chemical data, in particular, with NIR spectra. Nevertheless, FDA has not been applied to olive oil data.

Therefore, this work highlights that NIR spectra are particularly useful to estimate MUFAs fatty acids (in particular, oleic fatty acid). But the reliability or goodness of fit of all fatty acids predictions (SFAs, MUFA, PUFA, and for the disaggregated fatty acids: palmitic, stearic, palmitoleic, oleic, linolenic, and linolenic) can be improved by adding agro‐climatic data (specially, temperature and evapotranspiration) in the regression models. The high‐dimensional information contained in NIR spectra and agro‐climatic curves is summarized by using principal components analysis, where both scalar and functional approaches are used. The corresponding PCA and FPCA components are progressively introduced in regression models, whose goodness of fit is measured by DRMSE (dimensionless RMSE, useful in comparisons). The classical Kaiser's rule and the actual cross‐validation have been applied to determine the optimal number of components to be retained in the regression models (being obtained, in general, values around ten). The results show how the functional point of view and the use of both NIR and agro‐climatic information is better in the estimation of the fatty acids profile for a low number of components, the ideal situation to avoid the overfitting. Finally, as the International Olive Council (2012) establishes the characteristics of purity criteria for olive oils by using disaggregated fatty acids (see Table 1), DRMSE is depicted for palmitic, stearic, palmitoleic, oleic, linolenic, and linolenic fatty acids under the same previous assumptions. Although MUFAs estimations are, in general, the best, the disaggregated estimations for palmitoleic and oleic are different in reliability, being the last ones considerably better in goodness of fit.

## CONFLICT OF INTEREST

The authors declare no conflict of interest.

## ETHICAL STATEMENT

This study does not involve any human or animal testing.
