# Supplementary figures and images for: Functional approach and agro‐climatic information to improve the estimation of olive oil fatty acid content from near‐infrared data
Source: Food Sci Nutr. 2019 Dec 5;8(1):351–60. doi: 10.1002/fsn3.1312 (PMC6977507; doi:10.1002/fsn3.1312)

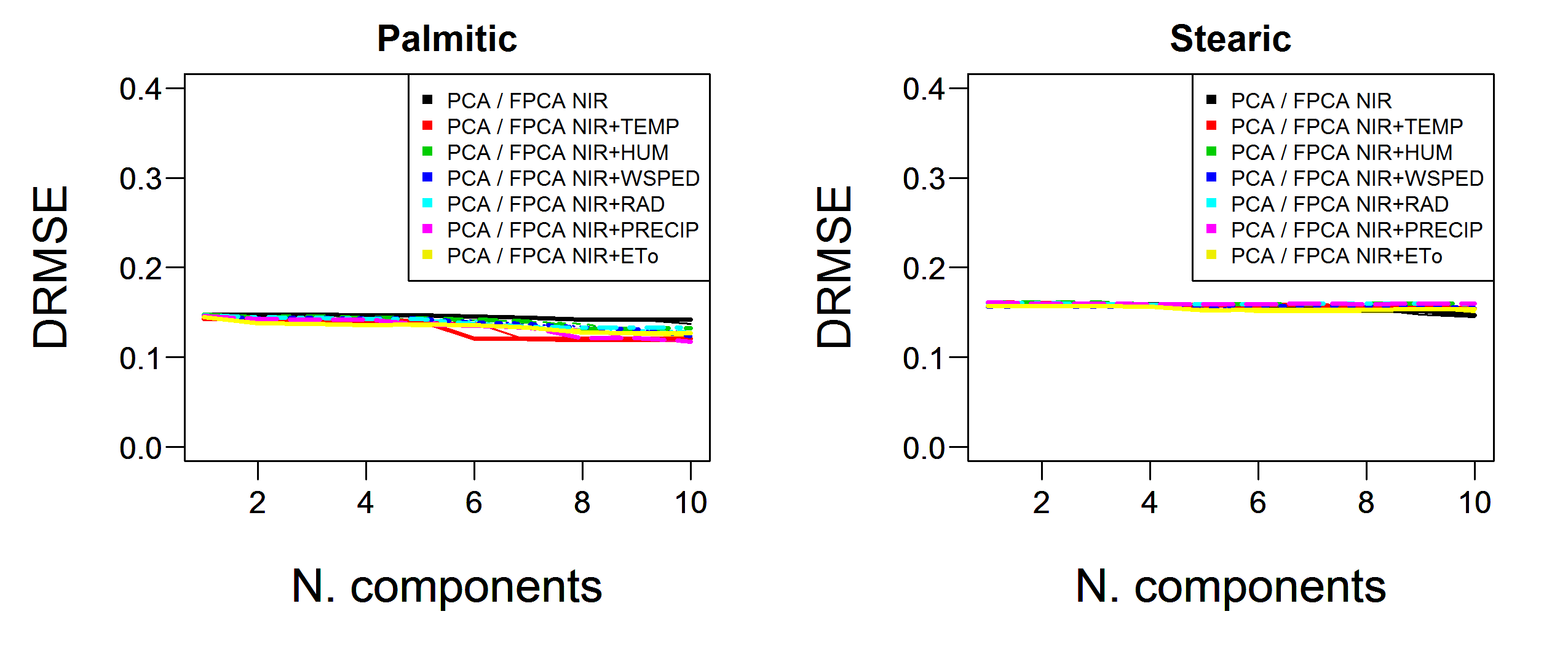

Supplement: Supplementary file 1 [file FSN3-8-351-s001.tiff]

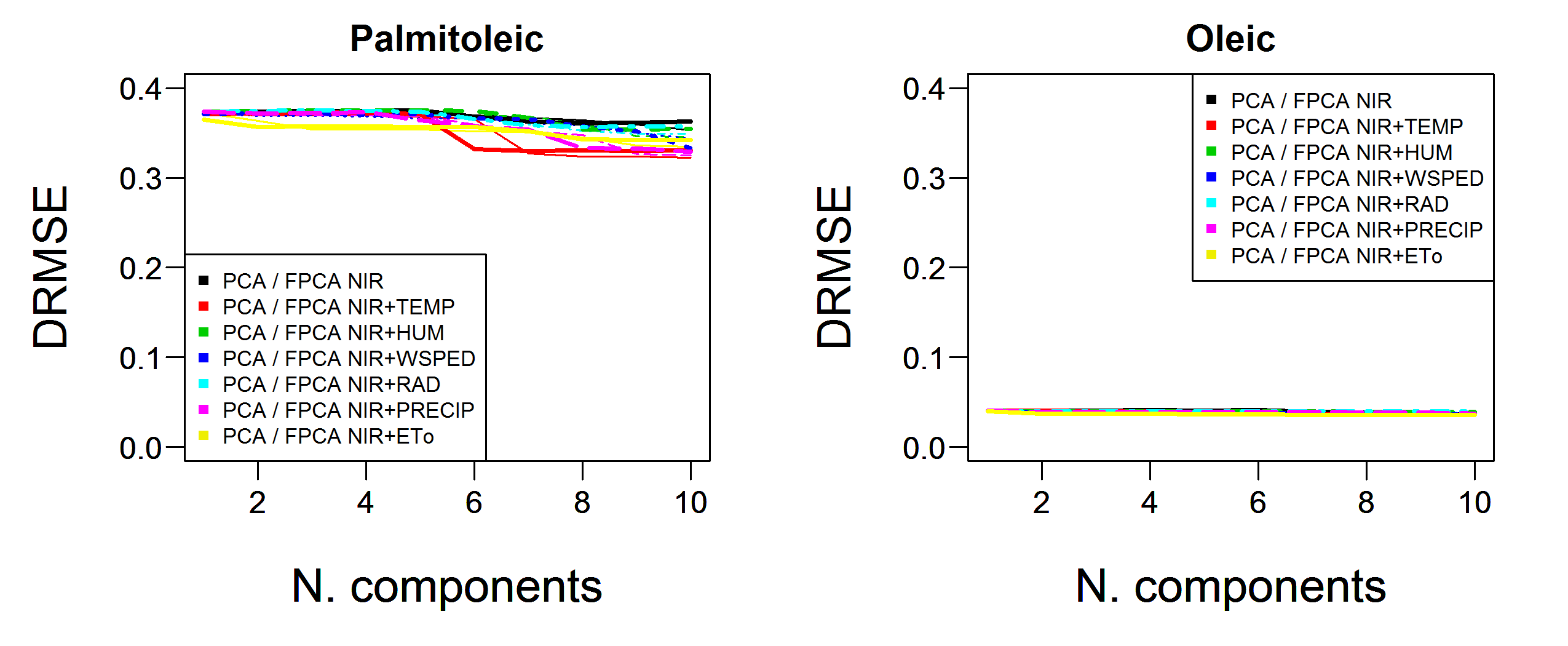

Supplement: Supplementary file 2 [file FSN3-8-351-s002.tiff]

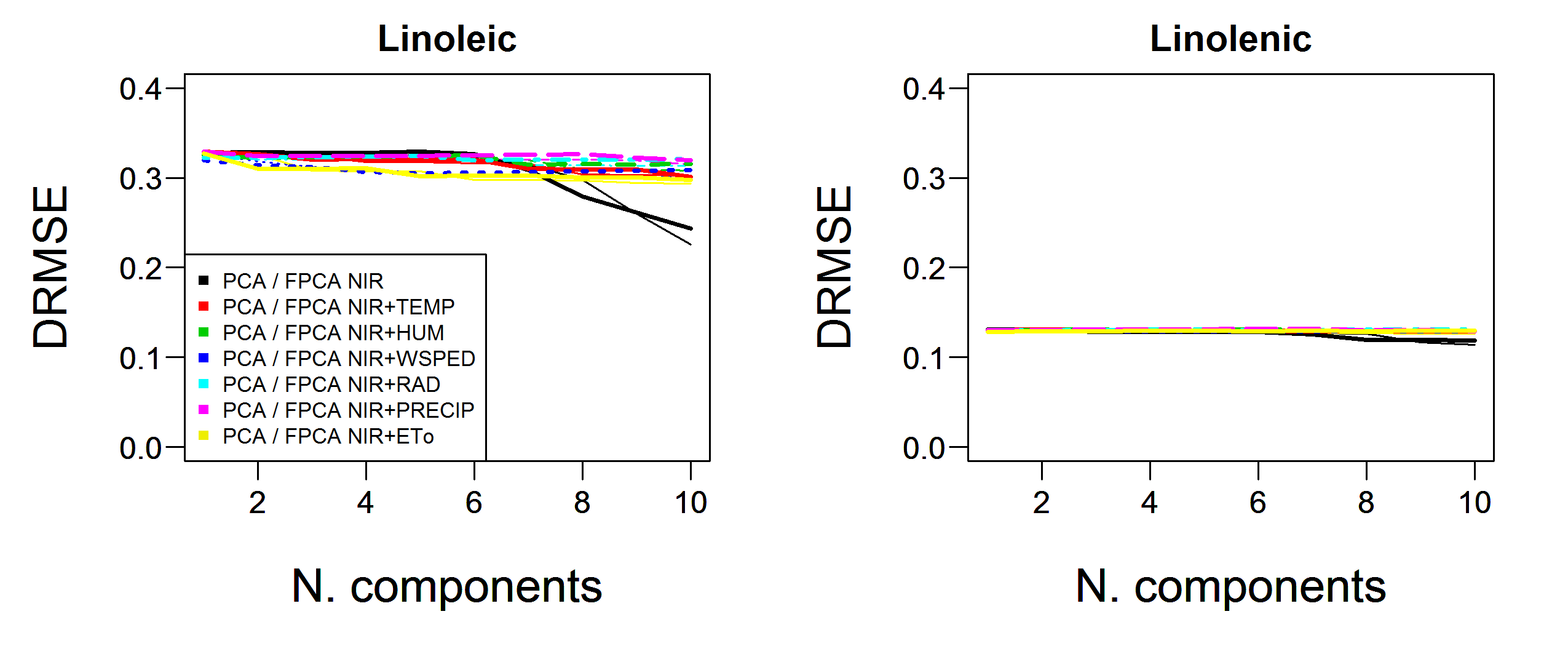

Supplement: Supplementary file 3 [file FSN3-8-351-s003.tiff]
